# Individual-Tree DBH Estimation from Airborne LiDAR Data Using MSFS–XGBoost

**DOI:** 10.3390/s26092873

**Published:** 2026-05-04

**Authors:** Pengfei Li, Yue Jia

**Affiliations:** Yunnan Provincial Mapping Institute, Kunming 650034, China; qazlipengfei@126.com

**Keywords:** airborne LiDAR, DBH estimation, Multi-Stage Feature Selection, machine learning

## Abstract

**Highlights:**

**What are the main findings?**

- A Multi-Stage Feature Selection (MSFS) framework integrating Pearson correlation, mutual information, and Boruta was developed to optimize high-dimensional airborne LiDAR point cloud features for individual-tree DBH estimation.
- The proposed MSFS–XGBoost model significantly improved prediction accuracy, achieving an $R^{2}$ of 0.901 and an RMSE of 1.647 cm, outperforming DTR, RFR, and GBM models.

**What are the implications of the main findings?**

- The proposed feature optimization strategy effectively reduces redundancy in LiDAR-derived features and enhances model stability for forest structural parameter estimation.
- The MSFS–XGBoost framework provides a reliable approach for accurate individual-tree DBH estimation and supports refined forest resource monitoring using airborne LiDAR data.

**Abstract:**

Diameter at breast height (DBH) is a fundamental structural parameter for forest inventory and ecological analysis. However, field-based measurements (e.g., diameter tape surveys) are labor-intensive and inefficient for large-scale applications. Airborne light detection and ranging (LiDAR) provides an efficient alternative for individual-tree DBH estimation. Nevertheless, LiDAR-derived features—defined as statistical descriptors of point cloud structure and radiometric properties—are typically high-dimensional and redundant, which may degrade model performance. To address this issue, this study proposes an integrated framework combining Multi-Stage Feature Selection (MSFS) and Extreme Gradient Boosting (XGBoost) for DBH estimation. A total of 104 variables, including LiDAR-derived features (height, density, intensity, and canopy structure metrics) and structural parameters (tree height, crown diameter, and crown area), were used as predictors. The MSFS framework was applied to progressively reduce feature redundancy and identify an optimal subset, which was then used to train the XGBoost model. The results demonstrate that the MSFS–XGBoost model achieved the best performance, with a coefficient of determination (R^2^) of 0.901 and a root mean square error (RMSE) of 1.647 cm. Compared with models using the original feature set, R^2^ increased by 0.384 and RMSE decreased by 1.146 cm. These findings indicate that the proposed framework effectively improves DBH estimation accuracy and provides a reliable approach for individual-tree parameter estimation and large-scale forest resource monitoring using airborne LiDAR data.

## 1. Introduction

Diameter at Breast Height (DBH) [1,2,3,4,5] is a fundamental structural parameter reflecting tree growth status, stand volume, and biomass, and it plays a central role in forest inventory, carbon stock assessment, and ecosystem modeling [6,7,8,9,10]. Traditional DBH measurements rely primarily on ground-based plot surveys. Although these methods provide high accuracy, they are constrained by terrain conditions, stand density, and low operational efficiency, making them inadequate for large-scale and rapid forest structural monitoring [11,12]. In recent years, with the rapid development of Light Detection and Ranging (LiDAR) technology, airborne LiDAR—characterized by high spatial resolution, strong canopy penetration capability, and high measurement efficiency—has become an important means of acquiring three-dimensional forest structural information. This technology enables the automated extraction and estimation of individual-tree structural parameters, such as tree height, crown width, and DBH.

Extensive research has been conducted worldwide on forest structural parameter estimation using airborne LiDAR. Early studies primarily employed empirical regression models, such as multiple linear regression, to establish DBH estimation equations based on extracted structural variables including tree height, crown diameter, and crown area [13,14,15,16]. Although these approaches are straightforward and easy to implement, their simple model structures limit their ability to capture complex nonlinear relationships among variables [17,18,19,20]. With advances in machine learning, increasing attention has been given to algorithms such as random forest, support vector regression, gradient boosting machines, and extreme gradient boosting [21,22,23,24]. These models generally outperform conventional regression approaches, such as multiple linear regression and stepwise regression, in terms of nonlinear fitting capability and generalization performance, leading to substantial improvements in parameter estimation accuracy [25,26]. In addition to these data-driven approaches, rule-based methods have also been explored for estimating forest structural parameters. These methods typically rely on geometric or structural assumptions derived from point cloud data to infer tree attributes [27]. However, their performance is often sensitive to point cloud completeness and structural complexity, which may limit their applicability in dense forest environments or when stem information is insufficient.

Despite these advances, LiDAR-derived point cloud features—representing statistical descriptors of three-dimensional structure and radiometric properties (e.g., height distribution metrics, canopy density indicators, return intensity statistics, and canopy structure metrics)—are typically high-dimensional, strongly correlated, and highly redundant. Directly incorporating such high-dimensional features into modeling processes may lead to issues such as the “curse of dimensionality,” overfitting, and model instability [28,29]. Consequently, feature selection and variable optimization have become critical factors influencing model performance.

Several studies have attempted to reduce feature redundancy and enhance model performance through methods such as correlation analysis, principal component analysis (PCA), and random forest-based feature importance ranking [30,31,32,33]. While these approaches can partially mitigate redundancy, single feature selection methods usually focus on linear relationships or one-dimensional importance, making it difficult to fully account for the coupling effects among multi-scale and multi-dimensional features [34,35]. In particular, at the individual tree level, airborne LiDAR point cloud features encompass diverse indicators related to height distribution, intensity statistics, canopy density, and morphological structure. The complex and heterogeneous relationships among these variables pose a major challenge for achieving efficient feature selection and optimal feature combinations, which remains a key scientific issue affecting DBH estimation accuracy.

To address these challenges, this study proposes an integrated DBH estimation framework based on Multi-Stage Feature Selection and Extreme Gradient Boosting (MSFS–XGBoost). The proposed approach first applies a Multi-Stage Feature Selection strategy that integrates Pearson correlation analysis, Mutual Information (MI), and the Boruta algorithm to perform feature screening and optimization from linear to nonlinear perspectives and from global to local scales. Subsequently, the selected optimal feature subset is used as input to an XGBoost model to construct a nonlinear regression relationship for accurate DBH estimation at the individual tree level. By combining feature optimization with ensemble learning, the proposed method effectively alleviates overfitting caused by feature redundancy and improves the robustness and interpretability of DBH estimation.

The main objectives of this study are as follows: (1) to establish an individual-tree feature extraction framework based on airborne LiDAR point cloud data, forming a multi-dimensional dataset of structural parameters and point cloud features; (2) to propose and validate the effectiveness of the Multi-Stage Feature Selection (MSFS) method for DBH estimation; and (3) to develop an integrated MSFS–XGBoost regression model and compare its performance with Decision Tree Regression (DTR), Random Forest Regression (RFR), and Gradient Boosting Machine (GBM), in order to investigate the impact of feature optimization on model accuracy and stability. The results of this study provide methodological support for the efficient utilization of airborne LiDAR data and to offer an effective technical approach for refined forest resource monitoring and structural parameter estimation.

## 2. Study Area and Data

This section describes the study area, data acquisition, and preprocessing procedures, providing the basis for subsequent analysis.

### 2.1. Study Area

**Figure 1:**
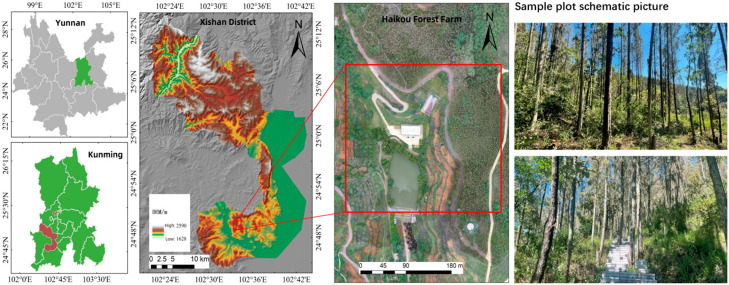
Location of the study area.

The study area is located in the Kuandiba forest region of Haikou Forest Farm, Xishan District, Kunming City, Yunnan Province, China (24°43′–24°56′ N, 102°28′–102°38′ E), as shown in Figure 1. The area is characterized by shallowly dissected mid-mountain terrain of the central Yunnan Plateau and belongs to the “lake plateau” geomorphological type. The region has a subtropical monsoon climate, with elevations ranging from approximately 1900 to 2200 m above sea level. The mean annual temperature is 14.6 °C, with a maximum of 34.4 °C and a minimum of −7.8 °C. The average annual precipitation is 909.7 mm, most of which occurs between June and July. Forest coverage in the study area is relatively high, at approximately 80.46%.

The dominant tree species include *Pinus armandii* Franch., *Pinus yunnanensis*, *Sabina chinensis*, *Cupressus lusitanica*, *Canarium oleosum*, *Alnus japonica* Steud., *Eucalyptus robusta* Smith, *Eucommia ulmoides* Oliver, and China fir, among others.

### 2.2. Data Acquisition

The airborne LiDAR data were acquired on 13 July 2023 using a Zhihang SF1650 six-rotor unmanned aerial vehicle (UAV) equipped with a South Surveying and Mapping SAL-1500 LiDAR sensor. During data acquisition, the flight altitude was set to 100 m, and the flight speed was maintained at 8 m/s. The forward overlap and side overlap of the flight lines were 80% and 70%, respectively. The resulting LiDAR point cloud had an average density of 226 points/m^2^. Detailed technical specifications of the UAV–LiDAR system are provided in Table 1.

**Figure 2:**
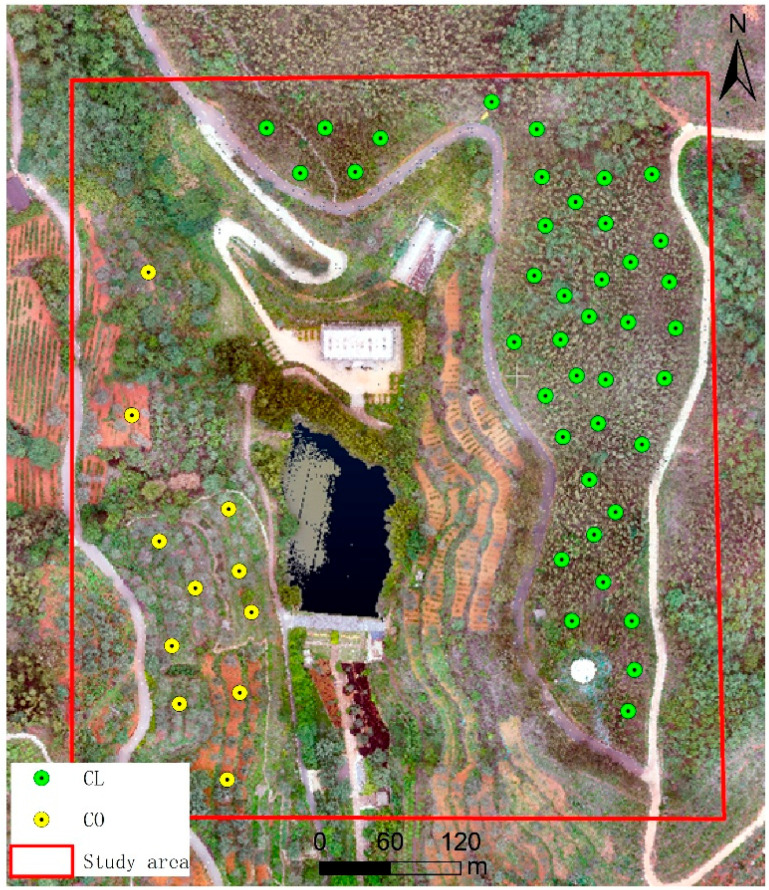
Distribution of ground survey plots in the study area.

During the field survey conducted in July 2023, a total of 51 randomly distributed circular plots with a radius of 10 m were established. Among them, 11 plots were classified as broadleaf forest plots (dominated by *Canarium oleosum*), and 40 plots were classified as coniferous forest plots (dominated by *Cupressus lusitanica* species), as shown in Figure 2.

During the ground survey, all individual trees with DBH ≥ 5 cm within each plot were located and measured. Tree positions were recorded using a Huace i90 RTK system, which provides a horizontal positioning accuracy of ±8 mm; during measurement, the survey pole was positioned as close as possible to the tree base. Individual-tree measurements were carried out using a diameter tape and a hypsometer, and information on tree species, DBH, tree height, and crown diameter was recorded. Summary statistics of forest structural parameters derived from the plot-level field survey are presented in Table 2.

### 2.3. Data Preprocessing

**Figure 3:**
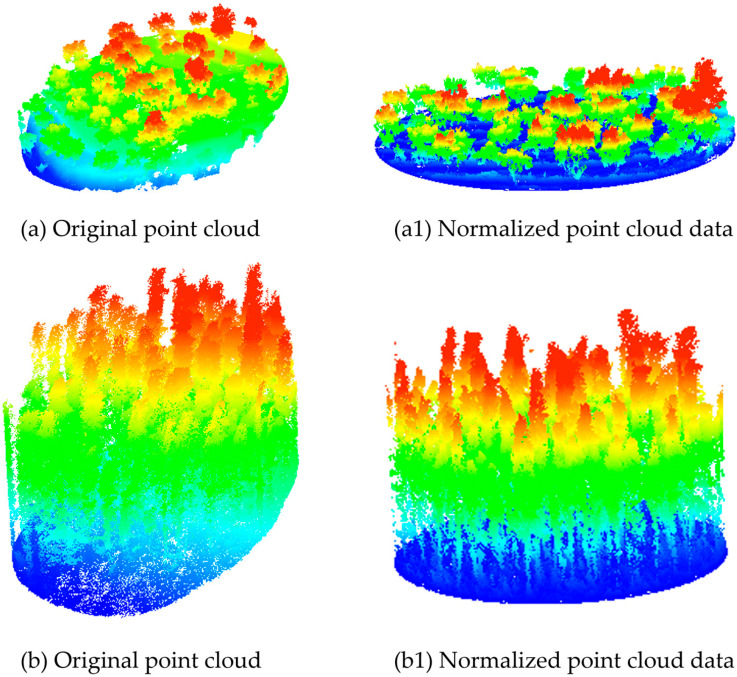
Illustration of raw and normalized point clouds in representative sample plots of the study area: (**a**) broadleaf forest; (**b**) coniferous forest.

To obtain accurate tree height information, the raw airborne LiDAR data were first subjected to noise removal [36]. Ground classification was then performed on the denoised point cloud data. In this study, the Cloth Simulation Filtering (CSF) algorithm was applied to separate ground points from vegetation points [37,38]. Based on the ground points extracted using CSF filtering, the mean elevation of LiDAR points within each grid cell was calculated. An Inverse Distance Weighting (IDW) interpolation method was subsequently employed to generate a Digital Elevation Model (DEM) with a spatial resolution of 0.5 m × 0.5 m. The DEM was then used to normalize the height information of discrete LiDAR returns by converting absolute elevation values into heights relative to the ground surface [39], resulting in a normalized point cloud (Figure 3). Finally, the normalized point cloud data corresponding to the selected sample plot extents were extracted for subsequent analysis.

## 3. Methods

### 3.1. Technical Workflow

**Figure 4:**
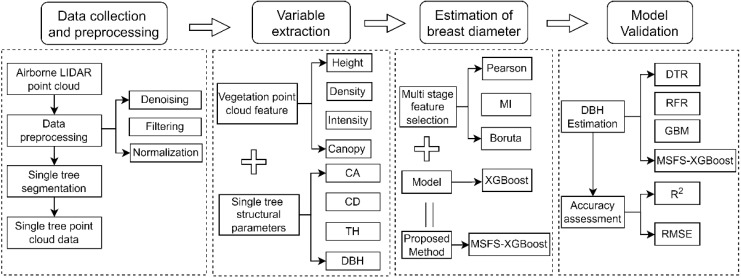
Workflow of the proposed MSFS–XGBoost framework. MSFS–XGBoost denotes the integration of Multi-Stage Feature Selection (MSFS) and Extreme Gradient Boosting (XGBoost). DTR, RFR, and GBM represent Decision Tree Regression, Random Forest Regression, and Gradient Boosting Machine, respectively. MI denotes Mutual Information. DBH is diameter at breast height, TH is tree height, CD is crown diameter, and CA is crown area. The “+” symbol represents the fusion of two feature sets; the “||” symbol indicates that the multi-stage feature selection and XGBoost are combined to form the proposed MSFS–XGBoost method.

Based on airborne Light Detection and Ranging (LiDAR) data and field-measured DBH data (used as target variables) from sample plots, this study developed an individual-tree DBH estimation framework integrating Multi-Stage Feature Selection and XGBoost ensemble regression, as illustrated in Figure 4. The workflow consists of four main components:

(1) Data acquisition and preprocessing: Raw airborne LiDAR point cloud data were subjected to noise removal, ground point filtering, and height normalization. Individual-tree point cloud data were then obtained using an individual tree segmentation algorithm.

(2) Variable extraction: Individual-tree-level point cloud feature variables were extracted using LiDAR360 software. These features were combined with three field-measured structural parameters to construct a 104-dimensional feature dataset. This component aims to provide standardized input variables for subsequent model development.

(3) DBH estimation: To address the high dimensionality and strong redundancy of point cloud features, a Multi-Stage Feature Selection (MSFS) method was proposed to progressively screen and optimize the initial feature set, reducing the feature dimensionality from 104 to 13. Based on the optimized feature subset, an XGBoost regression model was developed for individual-tree DBH estimation. In this study, DBH values were obtained from field measurements using a diameter tape and were used as target variables in the regression modeling process. DBH is therefore not directly measured from point cloud geometry, but estimated through a data-driven modeling approach that establishes relationships between field-measured DBH and LiDAR-derived features. Specifically, individual-tree structural parameters (e.g., tree height, crown diameter, and crown area) derived from point cloud segmentation were integrated with a set of point cloud-derived features, including canopy structure features, height distribution metrics, density-related features, and intensity-related features, to construct a comprehensive feature space for DBH estimation. This feature fusion strategy allows both explicit structural attributes and implicit point cloud characteristics to be jointly exploited, thereby enhancing the model’s ability to capture complex relationships with DBH.

(4) Model validation: Under identical feature conditions, Decision Tree Regression (DTR), Random Forest Regression (RFR), and Gradient Boosting Machine (GBM) models were constructed and compared with the proposed MSFS–XGBoost model. The coefficient of determination (R^2^) and root mean square error (RMSE) were used to comprehensively evaluate the estimation accuracy and stability of different models.

### 3.2. Individual Tree Segmentation

A Canopy Height Model (CHM) with a spatial resolution of 0.5 m was generated from the airborne LiDAR point cloud data of the study area. The CHM was obtained by subtracting the Digital Elevation Model (DEM) from the Digital Surface Model (DSM), i.e., CHM = DSM − DEM. The normalized point cloud data were then segmented based on the CHM, which was smoothed using a Gaussian filter with a sigma value of 0.7, where σ represents the standard deviation controlling the degree of smoothing. The spatial locations of individual-tree apexes identified from the CHM segmentation were used as seed points for individual tree segmentation using the Point Cloud Segmentation (PCS) algorithm. To reduce interference from understory vegetation, a minimum tree height threshold of 2 m was specified within the segmentation process. Specifically, only segmented objects with heights greater than 2 m were retained as valid trees, while objects below this threshold were excluded. This constraint helps to minimize the influence of low-height vegetation and improves the accuracy of individual-tree delineation.

Segmentation accuracy assessment: Segmentation accuracy was evaluated by comparing the detected individual-tree seed points with field-observed tree positions. Recall (*R*), Precision (*P*), and F-score (*F*) were calculated to assess the accuracy of individual tree segmentation [40]. The evaluation metrics were computed as follows:

(1) $$R=\frac{TP}{TP+FN}$$

(2) $$P=\frac{TP}{TP+FP}$$

(3) $$F=2\times \frac{R\times P}{R+P}$$

where *TP* represents the number of correctly segmented trees, *FN* denotes the number of omitted trees, and *FP* indicates the number of falsely segmented trees. A lower *R* value indicates more severe under-segmentation, whereas a lower *P* value reflects more pronounced over-segmentation.

### 3.3. Variable Extraction

The feature variables used for model development in this study consisted of individual-tree point cloud-derived features and field-measured structural parameters. Individual-tree point cloud features were extracted from the segmented point cloud data and categorized into four groups: canopy structure, height, density, and intensity, resulting in a total of 101 variables (Table 3). These LiDAR-derived features are statistical descriptors of the spatial distribution and radiometric properties of point clouds and have been widely adopted in forest parameter estimation studies [41,42,43]. Specifically, height-related features (e.g., percentiles, mean, and standard deviation) characterize the vertical structure of trees; density-related features describe the spatial distribution of points within the canopy; canopy structure metrics (e.g., canopy closure, leaf area index, and gap fraction) provide ecological information related to vegetation coverage and light penetration; and intensity-related features represent the amplitude of the returned LiDAR pulse, which is influenced by surface reflectance properties and acquisition geometry.

In addition, three field-measured individual-tree structural parameters—tree height, crown diameter, and crown area—were incorporated. These variables provide direct structural information and complement the LiDAR-derived features. Together, these variables formed a 104-dimensional feature dataset, providing a comprehensive representation of individual-tree structural and radiometric characteristics for subsequent DBH estimation modeling.

### 3.4. Multi-Stage Feature Selection

Because airborne LiDAR-derived point cloud features are typically high-dimensional and often exhibit strong redundancy and multicollinearity, directly incorporating them into modeling may introduce noise and reduce model accuracy and stability. To improve model robustness and generalization performance, this study proposes a Multi-Stage Feature Selection (MSFS) framework to systematically optimize the individual-tree feature set. The primary objectives of MSFS are to reduce feature dimensionality, enhance model generalization, avoid overfitting, and improve the interpretability of relationships between feature variables and DBH.

The MSFS framework integrates correlation analysis, information-theoretic measures, and feature importance evaluation, enabling progressive feature screening from linear to nonlinear relationships and from statistical relevance to model-based importance. This staged strategy is designed to gradually eliminate irrelevant and redundant features while preserving representative variables with strong explanatory power.

It should be noted that directly applying wrapper-based feature selection methods (e.g., Boruta) to the full high-dimensional feature set may lead to increased computational cost and a higher risk of overfitting, particularly under limited sample conditions. Therefore, a sequential filtering strategy was adopted in this study to first reduce the feature space using computationally efficient filter methods, followed by a wrapper-based optimization stage. This hybrid approach balances efficiency and effectiveness in high-dimensional feature selection.

However, it is acknowledged that such a sequential procedure may potentially discard features with weak individual correlations but strong joint contributions when interacting with other variables. This limitation should be considered when interpreting the results, and future studies may explore parallel or fully wrapper-based feature selection strategies to further enhance feature representation.

The MSFS procedure consists of the following three stages.

(1) Linear correlation screening

The Pearson correlation coefficient [44,45] was used to quantify the linear relationship between each feature variable and Diameter at Breast Height (DBH). The Pearson correlation coefficient is defined as:

(4) $$r=\frac{\sum \left(X_{i}-\bar{X}\right)\left(Y_{i}-\bar{Y}\right)}{\sqrt{\sum {\left(X_{i}-\bar{X}\right)}^{2}\sum {\left(Y_{i}-\bar{Y}\right)}^{2}}}$$

where $X_{i}$ and $Y_{i}$ represent the feature value and DBH value of the ith sample, respectively, and $\overset{ˉ}{X}$ and $\overset{ˉ}{Y}\;$ denote their corresponding means. The value of r ranges from −1 to 1, with a larger absolute value indicating a stronger linear relationship between the feature variable and DBH.

(2) Nonlinear correlation screening

Based on the results of linear correlation screening, Mutual Information (MI) [46] was further introduced to identify nonlinear dependency relationships between feature variables and DBH. Mutual information is defined as:

(5) $$I\left(X;Y\right)=\sum_{x\in X}\sum_{y\in Y}p\left(x,y\right)\log\frac{p\left(x,y\right)}{p\left(x\right)p\left(y\right)}$$

where p(x,y)  denotes the joint probability distribution of the feature variable and DBH, and p(x) and p(y) represent their marginal probability distributions. If the feature variable and DBH are statistically independent, I(X;Y)=0. Larger MI values indicate stronger dependency between the feature variable and DBH.

(3) Importance screening and feature optimization

To further identify features that make significant contributions to DBH estimation, the Boruta algorithm was employed for feature importance screening [47]. Boruta is a wrapper-based feature selection method built upon Random Forest (RF) models, and its core principle is to assess feature importance by comparing original features with randomly permuted shadow features [48].

In this study, shadow features are generated by randomly shuffling the values of each original feature across samples, thereby destroying any real association with the target variable while preserving the original data distribution. These shadow features serve as a baseline to evaluate whether the importance of a real feature is significantly higher than that of noise. The Boruta procedure consists of the following steps:

a. Creating shadow features by randomly shuffling each original feature;

b. Training an RF model and calculating importance scores for both original and shadow features;

c. Comparing the importance of each original feature with the maximum importance among the shadow features;

d. Retaining features that are significantly more important than shadow features and removing otherwise;

e. Repeating the process iteratively until the feature selection results converge.

### 3.5. Model Construction

(1) Integrated MSFS–XGBoost modeling

To achieve an effective integration of feature selection and regression modeling, an integrated DBH estimation framework based on Multi-Stage Feature Selection and Extreme Gradient Boosting (MSFS–XGBoost) was developed in this study. The proposed framework aims to efficiently exploit high-dimensional point cloud features and achieve accurate DBH prediction through progressive feature optimization combined with ensemble regression modeling.

The optimal feature subset generated by the MSFS module was used as the input feature matrix for the XGBoost model. XGBoost employs Classification and Regression Trees (CART) as weak learners and iteratively fits residuals through a gradient boosting strategy to predict DBH. Model training and error optimization were achieved by minimizing the following objective function [49]:

(6) $$Obj=\sum_{i=1}^{n}[l(y_{i},{{\hat{y}}_{i}}^{\left(t-1\right)}+f_{t}\left(x_{i}\right))]+Ω\left(f_{t}\right)$$

where n is the number of training samples, $x_{i}$ represents the feature vector of the i-th sample, and $y_{i}$ denotes the corresponding observed DBH value. ${{\hat{y}}_{i}}^{\left(t-1\right)}$ is the predicted value of the *i*-th sample at the (t − 1)-th iteration, and $f_{t}\left(x_{i}\right)$ represents the prediction of the t-th regression tree for sample $x_{i}$.

Here, t denotes the iteration index corresponding to the *t*-th tree in the additive model. The function l(⋅) denotes the loss function, for which the squared error loss is adopted in this study. $\Omega (f_{t})$ represents the regularization term that penalizes model complexity and helps prevent overfitting. The regularization term is defined as:

(7) $$Ω\left(f_{t}\right)=\gamma T+\frac{1}{2}\lambda \sum_{j=1}^{T}\omega_{j}^{2}$$

where T is the number of leaf nodes, $\omega_{j}$ denotes the weight of the j th leaf node, and γ and λ are the split penalty parameter and weight regularization coefficient, respectively.

The integrated MSFS–XGBoost modeling procedure consists of four stages:

(a) Input preparation: individual-tree point cloud feature variables and field-measured DBH values were extracted;

(b) Multi-Stage Feature Selection (MSFS): Pearson correlation analysis, Mutual Information (MI), and the Boruta algorithm were jointly applied to perform feature dimensionality reduction and optimization, yielding an optimal feature subset;

(c) XGBoost modeling: the optimized feature subset was used to train the XGBoost model, with the loss function iteratively minimized through gradient boosting;

(d) Model output and validation: DBH predictions were generated, and model performance was evaluated using R^2^ and RMSE.

During node splitting, XGBoost calculates the feature split gain (Gain) to determine the optimal split point, which is defined as:

(8) $$G_{ain}=\frac{1}{2}\left[\frac{G_{L}^{2}}{H_{L}+\lambda }+\frac{G_{R}^{2}}{H_{R}+\lambda }+\frac{{\left(G_{L}+G_{R}\right)}^{2}}{H_{L}+H_{R}+\lambda }\right]-\gamma$$

$G_{L}$ and $G_{R}$ denote the sums of first-order gradients in the left and right child nodes, respectively, where the first-order gradient is defined as the derivative of the loss function with respect to the model prediction. $H_{L}$ and $H_{R}$ represent the corresponding sums of second-order gradients, which are the second derivatives of the loss function and capture the curvature information of the objective function. λ is the L2 regularization parameter used to control the complexity of leaf weights and reduce overfitting, while γ is the minimum loss reduction required for a node split, serving as a penalty term to control model complexity and prevent unnecessary tree growth. A larger Gain value indicates a greater contribution of the corresponding feature to model splitting.

By integrating MSFS with XGBoost, the proposed framework achieves end-to-end optimization from feature dimensionality reduction to nonlinear regression modeling. This integrated strategy not only reduces redundancy in input features but also improves model stability and interpretability. Compared with conventional modeling approaches based on single-stage feature selection, the MSFS–XGBoost framework enables more effective utilization of point cloud feature information and maintains high prediction accuracy and generalization capability under limited sample conditions.

(2) Selection of comparison models

To evaluate the performance of the proposed MSFS–XGBoost integrated regression framework, three representative decision tree-based machine learning models were selected for comparative analysis (Table 4). These models form a hierarchical system ranging from a single-tree structure to different ensemble learning strategies, enabling a systematic assessment of the effects of feature optimization on model accuracy and stability.

This progressive model selection strategy—from simple to more complex architectures—ensures model interpretability while allowing a robust evaluation of the effectiveness and stability of the MSFS–XGBoost integrated regression approach.

All models were implemented using the Python scikit-learn library. Specifically, DecisionTreeRegressor, RandomForestRegressor, and GradientBoostingRegressor from scikit-learn, as well as XGBRegressor from the XGBoost library, were employed to construct DBH estimation models [53].

(3) Model parameter optimization

During model training, to minimize potential bias introduced by subjective hyperparameter settings, a Grid Search strategy was adopted to optimize the hyperparameters of all four models. Grid search systematically explores all possible parameter combinations within predefined ranges using an exhaustive search strategy and selects the parameter set that yields the best model performance as the optimal configuration [54].

(4) Model evaluation metrics

Model performance was evaluated using the coefficient of determination (R^2^) and the root mean square error (RMSE). A higher R^2^ value and a lower RMSE value indicate superior model performance. The evaluation metrics were calculated as follows:

(9) $$R^{2}=1-\frac{\sum_{i=1}^{n}{\left(y_{i}-{\hat{y}}_{i}\right)}^{2}}{\sum_{i=1}^{n}{\left(y_{i}-\bar{y}\right)}^{2}}$$

(10) $$RMSE=\sqrt{\frac{1}{n}\sum_{i=1}^{n}{\left(y_{i}-{\hat{y}}_{i}\right)}^{2}}$$

where n  is the number of samples, $y_{i}\;$ represents the observed DBH value, ${\hat{y}}_{i}$ denotes the predicted DBH value, and $\overset{ˉ}{y}$ is the mean of the observed DBH values.

## 4. Results

### 4.1. Results of Individual Tree Segmentation

The forests in the study area can be broadly classified into broadleaf forest stands and coniferous forest stands. Due to substantial differences in crown size between these two forest types, individual tree segmentation was performed separately for stands with different crown scales. The results of individual tree segmentation are presented in Table 5.

The correctly segmented individual trees were validated by comparison with field-measured tree data, and samples that best matched the field observations were selected. Based on the segmentation results, individual-tree point cloud data and corresponding individual-tree structural parameters, including tree height, crown diameter, and crown area, were ultimately extracted for subsequent analysis.

By extracting tree height, crown diameter, and crown area from correctly segmented individual-tree point clouds and establishing regression relationships with corresponding field-measured data, the extraction accuracy of these structural parameters was evaluated. The results indicate that tree height derived from point cloud data exhibited high accuracy, while the extracted crown diameter was slightly larger than the field-measured values, with a strong correlation observed between the two.

**Figure 5:**
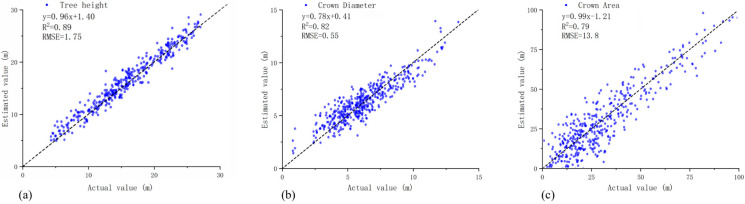
Regression relationships between extracted individual-tree parameters and field-measured values. The dashed line represents the 1:1 reference line. (**a**) Tree height; (**b**) Crown diameter; (**c**) Crown area.

Compared with tree height and crown diameter, crown area exhibited relatively lower extraction accuracy and a larger dispersion in the regression relationship. This discrepancy is likely associated with uncertainties in individual-tree segmentation, particularly at crown boundaries, where overlapping canopies and closely spaced neighboring trees may lead to over-segmentation or boundary ambiguity (Figure 5). Overall, the extraction accuracy of tree height, crown diameter, and crown area was acceptable and demonstrates good application potential, supporting their use as input variables for DBH estimation models.

### 4.2. Results of Feature Selection

The feature variables used in this study consisted of three canopy structure variables, 46 height-related variables, 10 density-related variables, 42 intensity-related variables, and three field-measured structural parameters, resulting in a total of 104 variables. The proposed Multi-Stage Feature Selection (MSFS) method was applied to the extracted feature dataset to perform progressive feature screening, and three feature subsets were obtained at different stages, as summarized in Table 6.

**Figure 6:**
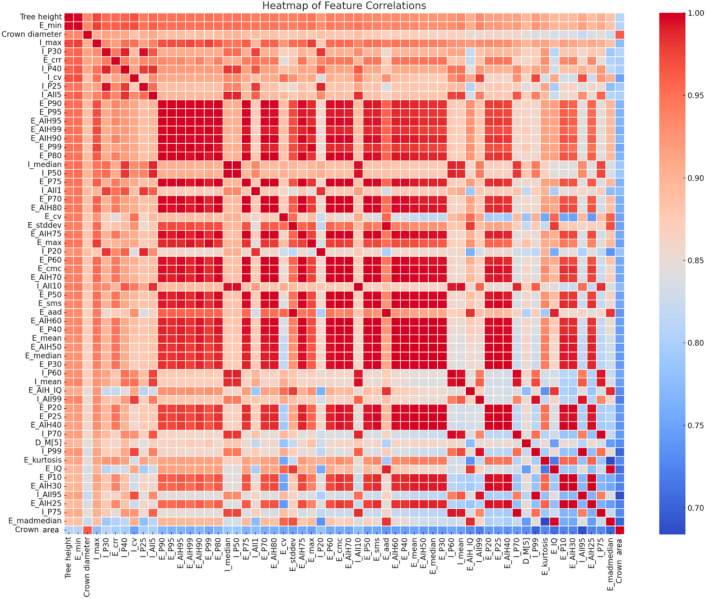
Pearson correlation heatmap between feature variables and DBH.

In the Pearson correlation stage, features with weak linear relationships to DBH (|r| < 0.3) were removed. As a result, 60 features were retained, including 38 height-related variables, 18 intensity-related variables, 1 density-related variable, and 3 structural parameters, while 44 features were eliminated from the original feature set. The retained features generally exhibited relatively strong linear correlations with DBH and covered multiple aspects of forest structure, including height, intensity, density, and canopy characteristics, thereby ensuring comprehensive representation of the feature space. Although certain groups of similar features (e.g., height percentiles) still showed some degree of clustering, feature redundancy was substantially reduced compared with the original high-dimensional feature set (Figure 6).

Following optimization at the Pearson correlation stage, Mutual Information (MI) values between the retained feature variables and Diameter at Breast Height (DBH) were further calculated to capture nonlinear dependency relationships. The results show that the overall distribution of MI values was right-skewed, with only a limited number of variables exhibiting high information contribution. Among them, tree height had the highest MI value, indicating its most significant contribution to DBH estimation, which is consistent with established principles of tree growth.

**Figure 7:**
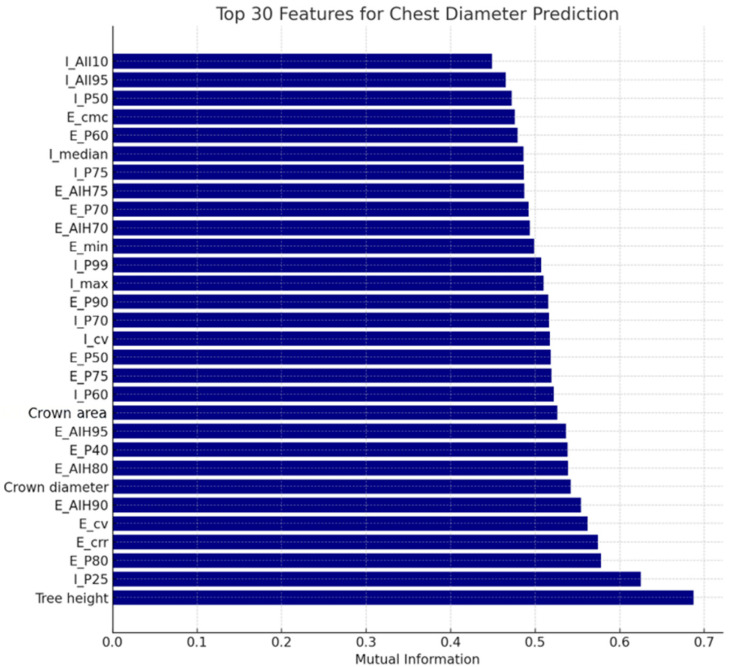
Distribution of mutual information values between feature variables and DBH.

To ensure objectivity in feature selection, a distribution-based dynamic threshold was adopted, defined as the mean MI value plus one standard deviation (μ + σ), where μ and σ denote the mean and standard deviation of the MI values, respectively. In this study, this threshold was approximately 4.5, and variables with MI > 4.5 were retained (Figure 7). This threshold effectively distinguishes high-information features from background noise features, ensuring that the retained variables possess strong explanatory power and meaningful nonlinear associations with DBH.

Finally, the Random Forest-based Boruta algorithm was applied to the candidate feature set obtained from the previous stage to perform feature importance evaluation and optimization. By comparing original features with randomly generated shadow features, the Boruta algorithm automatically identifies variables that are statistically significant in terms of importance. After multiple iterations, Boruta adaptively determined an optimal feature subset, in which structural parameters, height-related variables, and intensity-related variables were all represented.

The final selected feature set consisted of 13 variables (Table 7), representing a substantial reduction in dimensionality compared with the initial 104-dimensional feature set, while retaining the key information required for accurate DBH estimation.

The retained feature set includes structural parameters, height-related variables, and intensity-related variables, indicating that DBH is influenced by multiple aspects of tree structure. Structural parameters (e.g., tree height and crown size) and height-related features provide direct and indirect descriptions of tree size and vertical structure, which are closely associated with DBH. Notably, several intensity-related features (e.g., I-max, I-P99, I-cv, I-AII95, and I-AII10) were also retained, suggesting their potential relevance in DBH estimation. These features characterize the magnitude and variability of LiDAR return intensity and may indirectly reflect differences in canopy structure and internal heterogeneity among trees. This result highlights that, in addition to geometric attributes, radiometric information derived from LiDAR data can also contribute to DBH estimation.

### 4.3. Individual-Tree DBH Estimation

A total of 600 individual-tree samples were selected from the sample plots, of which 400 trees (66.7%) were used for model training and 200 trees (33.3%) for model validation. In addition, a 5-fold cross-validation strategy was employed as a complementary evaluation approach to assess the robustness and generalization ability of the models. The dataset consisted of 500 coniferous trees (*Cupressus lusitanica*) and 100 broadleaf trees (*Canarium oleosum*). Summary statistics of the individual-tree samples are provided in Table 8.

Based on the different feature subsets obtained using the MSFS procedure, the XGBoost model was used to estimate individual-tree DBH and its performance was compared with that of a single-tree model (DTR), a bagging-based ensemble model (RFR), and a boosting-based ensemble model (GBM). Model hyperparameters were optimized using a grid search strategy, and model performance was evaluated using the coefficient of determination (R^2^) and the root mean square error (RMSE).

The results show that when the original 104-dimensional feature set was directly used for modeling, the overall estimation accuracy of all models was relatively low (Table 9). Among them, the Decision Tree Regression (DTR) model exhibited the poorest performance, with an R^2^ value of 0.330 and an RMSE of 4.121, indicating its limited ability to capture complex nonlinear relationships between DBH and high-dimensional point cloud features.

The ensemble learning models showed moderate improvements over DTR; however, the performance gains were relatively limited. Both RFR and GBM achieved higher estimation accuracy than DTR but remained constrained by the influence of high-dimensional redundant features. In comparison, XGBoost achieved the best estimation performance when using the original feature set, with R^2^ values of 0.558 and 0.517 for the training and validation datasets, respectively, highlighting its advantages in modeling feature interactions and nonlinear relationships. Nevertheless, due to the absence of feature selection and optimization, the model was still affected by feature noise and redundancy, and its overall predictive performance was not fully realized.

These results indicate that high-dimensional redundant features can substantially limit DBH estimation performance, thereby providing a clear justification for introducing Multi-Stage Feature Selection (MSFS) to optimize the feature space in subsequent analyses.

As the feature set was progressively optimized, the performance of the XGBoost model improved substantially. When using the 60-dimensional feature subset retained after Pearson correlation screening, the overall R^2^ values increased markedly, with boosting-based models showing the most pronounced performance gains. Specifically, the R^2^ of the XGBoost model increased to 0.679 (an improvement of 0.162 compared with the original feature set), while the RMSE decreased to 2.389, indicating that removing features weakly correlated with DBH effectively enhanced the model’s ability to capture key predictive information. In contrast, DTR and RFR exhibited relatively limited improvements at this stage and remained more susceptible to data noise.

After further reducing the feature dimensionality to 30 variables using the Mutual Information (MI) method, performance differences among the models became more pronounced. The R^2^ of the XGBoost model increased to 0.820, while that of GBM also improved to 0.619, demonstrating that MI-based screening effectively eliminated redundant features and enhanced model accuracy. However, the performance of RFR and DTR remained comparatively lower, highlighting their limitations in utilizing optimized feature sets.

The 13-dimensional feature subset selected using the Boruta algorithm yielded the best overall model performance. The ranking of model accuracy was XGBoost > GBM > RFR > DTR. Among them, the proposed MSFS–XGBoost model achieved the highest accuracy, with an R^2^ of 0.901 and an RMSE of 1.647. Compared with the model built using the original 104-dimensional feature set, R^2^ increased by 0.384 and RMSE decreased by 1.146. Under the same feature conditions, the GBM model achieved an R^2^ of 0.783 with an RMSE of 2.049, while the RFR model yielded an R^2^ of 0.693 and an RMSE of 3.248. The DTR model showed the lowest prediction accuracy (R^2^ = 0.553), although its R^2^ still improved by 0.223 compared with the model using the original feature set.

**Figure 8:**
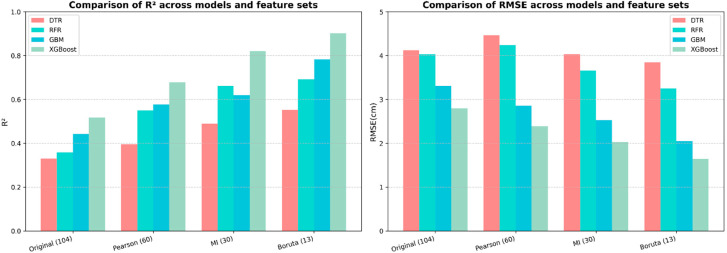
Accuracy comparison of different models under different feature sets.

Overall, these results indicate that, when combined with Multi-Stage Feature Selection, XGBoost can more effectively exploit optimized feature information, resulting in superior predictive performance for individual-tree DBH estimation. A comparison of model accuracy across different feature sets is shown in Figure 8.

Overall, the quality of input features plays a critical role in the performance of DBH estimation models. The unscreened high-dimensional feature set contains substantial redundant information, which to some extent constrains the model’s ability to learn effective predictive patterns. In contrast, after progressively optimizing the feature space through the Multi-Stage Feature Selection (MSFS) procedure, model prediction performance was significantly improved.

Among all evaluated models, the proposed MSFS–XGBoost framework consistently achieved the best performance across different feature set stages. Under the final optimized feature subset, its coefficient of determination (R^2^) increased by 0.384 compared with the model using the original feature set, indicating a strong capability to exploit optimized features and maintain stable learning performance. Overall, comparative results demonstrate that, under the conditions of this study, the MSFS–XGBoost model achieved the most accurate DBH estimation.

To further evaluate the robustness of the models, a 5-fold cross-validation was conducted using the optimal 13-dimensional feature set. The results are presented in Table 10.

The MSFS–XGBoost model achieved the highest accuracy, with an average R^2^ of 0.881 (±0.016) and RMSE of 1.677 (±0.031), followed by GBM, RFR, and DTR. The relatively low standard deviations indicate that all models exhibit stable performance across different data partitions.

Moreover, the relative performance ranking of the models (XGBoost > GBM > RFR > DTR) is consistent with the results obtained from the hold-out validation. This consistency further demonstrates that the proposed MSFS–XGBoost framework is robust and not sensitive to data partitioning.

### 4.4. Model Evaluation and Validation

(1) Method validation

To further evaluate the effectiveness of the proposed MSFS framework, a comparative experiment was conducted using LASSO as a baseline feature selection method. LASSO performs feature selection through L1 regularization, where the optimal subset of features is automatically determined by minimizing prediction error via cross-validation.

For consistency, the features selected by LASSO were used as inputs to the same XGBoost model with identical parameter settings. The comparison results are summarized in Table 11.

As shown in Table 11, the MSFS-based feature subset achieved better performance than LASSO, with a higher coefficient of determination (R^2^ = 0.901) and lower root mean square error (RMSE = 1.647 cm), compared to R^2^ = 0.814 and RMSE = 2.219 cm for LASSO. Notably, LASSO retained a larger number of features (20 variables), whereas MSFS selected only 13 features, indicating that improved prediction accuracy is primarily attributed to feature relevance rather than feature quantity.

It should be noted that the number of features selected by LASSO is not predefined but depends on the regularization parameter, which is optimized to achieve the best predictive performance. Therefore, the comparison reflects the optimal configuration of each method rather than enforcing an identical feature dimension. The inferior performance of LASSO may be related to its reliance on linear assumptions, which limits its ability to capture complex nonlinear relationships. In contrast, the proposed MSFS framework integrates multiple selection strategies, enabling more effective identification of informative features in high-dimensional LiDAR data.

(2) Model Evaluation

Based on the identified optimal feature subset and the optimized model parameters, 40% of the individual-tree samples were randomly selected to construct four models and to evaluate their adaptability across the two dominant tree species (Table 12). The results indicate that XGBoost achieved the best performance for both *Cupressus lusitanica* and *Canarium oleosum*, with R^2^ values of 0.905 and 0.896, and RMSE values of 1.518 and 1.614, respectively. These results demonstrate its strong fitting capability and robust cross-species generalization performance.

The GBM model achieved R^2^ values of 0.813 for *Cupressus lusitanica* and 0.782 for *Canarium oleosum*, outperforming RFR and DTR for both species. However, due to differences in algorithmic structure, its overall accuracy remained slightly lower than that of XGBoost. The RFR model exhibited lower accuracy than GBM and XGBoost, but its ensemble voting mechanism still resulted in better performance than the single decision tree model, with R^2^ values of 0.675 and 0.691 for *Cupressus lusitanica* and *Canarium oleosum*, respectively.

In contrast, the DTR model showed the lowest prediction accuracy among the four models for both tree species, reflecting its limited capacity to capture complex relationships among high-dimensional features due to its relatively simple structure.

**Figure 9:**
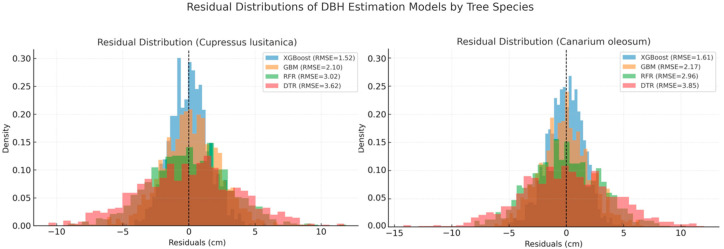
Residual distributions of DBH estimation models for *Cupressus lusitanica* (**left**) and *Canarium oleosum* (**right**).

Further comparison of the predictive performance of the four models was conducted based on the distribution of residuals (Figure 9). In the *Cupressus lusitanica* plots, the XGBoost model exhibited the most concentrated residuals, with the majority falling within ±2 cm, indicating minimal deviation between predicted and measured DBH values. The GBM model showed a slightly broader residual distribution but still maintained relatively good stability. In contrast, the RFR and DTR models displayed more dispersed residuals, with a higher occurrence of large prediction errors, reflecting their limited capacity to capture complex relationships among high-dimensional features.

In the *Canarium oleosum* plots, the residual distributions of all models were generally more dispersed than those observed in the coniferous plots, suggesting that the increased structural complexity of broadleaf trees poses greater challenges for DBH estimation. Nevertheless, the relative performance patterns among the models were consistent with those observed in the *Cupressus lusitanica* plots. The XGBoost model continued to exhibit the most concentrated residual distribution, demonstrating superior prediction stability compared with the other three models.

**Figure 10:**
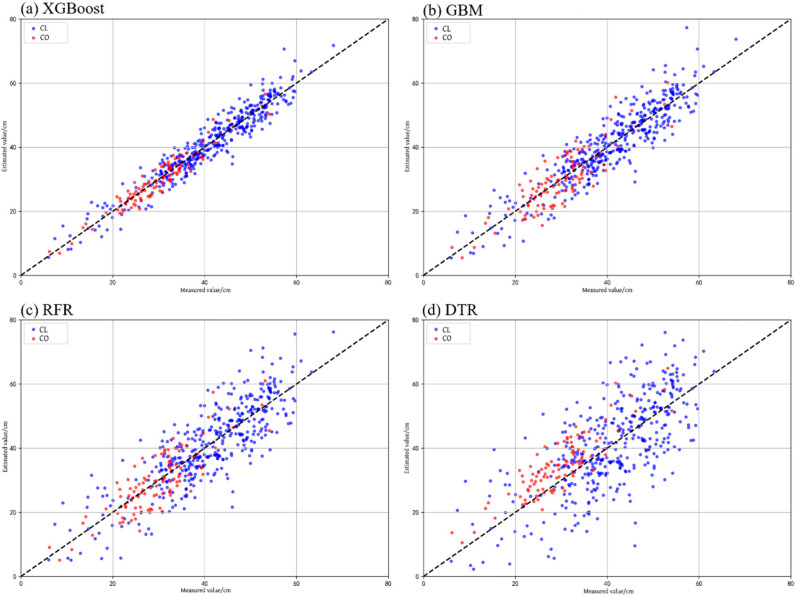
Scatter plots of measured versus estimated DBH obtained using four regression models. The dashed line represents the 1:1 reference line.

Based on the overall characteristics of the residual distributions, the XGBoost model consistently exhibited higher estimation accuracy and greater stability for both tree species when using the MSFS-optimized feature set. Furthermore, samples of *Cupressus lusitanica* and *Canarium oleosum* were separately input into the four models using their respective optimal parameter settings and feature subsets. Scatter plots of measured versus estimated DBH values were then generated (Figure 10) to visually compare the fitting performance of the different models.

## 5. Discussion

The Multi-Stage Feature Selection (MSFS) framework proposed in this study effectively mitigates the redundancy and instability introduced by high-dimensional features in individual-tree DBH estimation. Airborne LiDAR point cloud data provide rich spatial and radiometric information related to forest structure; however, such high-dimensional feature spaces are often characterized by strong multicollinearity and redundancy. When unfiltered features are directly used as model inputs, they not only increase model complexity but also tend to induce overfitting and unstable prediction performance [55]. By integrating Pearson correlation analysis, Mutual Information (MI), and the Boruta algorithm, the MSFS framework progressively eliminates irrelevant or weakly related variables and reduces the original feature set from 104 variables to a compact subset of 13 representative features. The results demonstrate that the optimized feature set preserves key structural information while substantially compressing the feature space and reducing redundancy.

It is important to note that airborne LiDAR data generally provide limited direct information on tree stem geometry, particularly in the mid-story layer, due to occlusion effects and canopy shielding. As a result, DBH cannot be directly measured from airborne point clouds. In this study, DBH is estimated indirectly through regression modeling by exploiting statistical relationships between field-measured DBH and LiDAR-derived features. These features, including tree height, crown dimensions, canopy structure metrics, and intensity-related variables, serve as proxy indicators that are functionally related to tree size and growth conditions. This indirect estimation strategy is consistent with previous studies [56], which have demonstrated that although stem information is sparse in airborne LiDAR data, structural and radiometric features can still provide sufficient predictive power for DBH estimation when combined with robust machine learning models.

From a methodological perspective, the MSFS framework follows a hierarchical and progressive optimization strategy. Pearson correlation analysis efficiently identifies features with weak linear relationships to DBH, thereby providing a concise candidate feature set for subsequent screening. The MI stage further captures nonlinear dependencies between features and DBH, compensating for the limitations of linear correlation-based methods. Finally, the Boruta algorithm evaluates feature importance using random forest ensembles, offering a statistically robust and global assessment of feature contributions. Together, these three stages constitute a “linear screening–nonlinear identification–global validation” feature optimization pipeline. However, it should be noted that this sequential selection strategy may introduce a potential limitation: features that exhibit weak individual linear or nonlinear relationships with DBH may still contribute significantly through interactions with other variables, and could therefore be excluded during the early screening stages.

In this study, this risk is partially mitigated by incorporating the Boruta algorithm, which evaluates feature importance in a multivariate and model-based context. Nevertheless, since Boruta is applied only to the reduced feature subset, it cannot fully recover features eliminated in earlier stages. This reflects a trade-off between computational efficiency and exhaustive feature exploration. Given the high dimensionality and redundancy of LiDAR-derived features, the progressive filtering strategy adopted in MSFS helps improve feature quality and model stability, which is also supported by the observed performance improvements. Future work could further explore hybrid or parallel feature selection strategies to better capture complex feature interactions. Similar conclusions were reported by Zhang L.X. et al. [57], who showed that appropriate feature optimization can significantly enhance the accuracy of LiDAR-based forest parameter estimation. Overall, MSFS not only achieves effective dimensionality reduction but also provides a more stable and informative feature space for subsequent modeling.

At the model level, this study systematically compared three representative decision tree-based regression models, namely Decision Tree Regression (DTR), Random Forest Regression (RFR), and Gradient Boosting Machine (GBM). The results reveal a clear hierarchical pattern in model performance, corresponding to increasing algorithmic complexity and feature optimization. The single-tree DTR model, while advantageous in terms of interpretability, is highly sensitive to noise and feature redundancy, resulting in relatively low prediction accuracy. RFR improves model stability through a bagging ensemble strategy, yet its ability to capture complex interactions among features remains limited. GBM introduces a residual-based boosting mechanism that enhances nonlinear fitting capability; however, its performance is strongly influenced by hyperparameters such as learning rate and tree depth.

In contrast, the XGBoost model achieved the best performance when combined with the MSFS-optimized feature set, with an R^2^ of 0.901 and an RMSE of 1.647, significantly outperforming the other three models. This superior performance can be attributed to XGBoost’s incorporation of regularization terms into the objective function, as well as its efficient feature-splitting strategy and parallel tree construction mechanism. These characteristics enable XGBoost to enhance fitting capacity while effectively suppressing overfitting. Similar findings were reported by Hu T. et al. [58,59], who demonstrated that XGBoost generally outperforms traditional random forest models in forest structural parameter estimation tasks involving high-dimensional features and complex sample distributions, further confirming its strong generalization ability.

The proposed MSFS–XGBoost integrated framework highlights the synergistic benefits of feature optimization and ensemble learning. In conventional modeling workflows, feature selection and model construction are often treated as independent stages, with limited interaction between them. In contrast, the MSFS–XGBoost framework performs feature dimensionality reduction and relevance optimization at the input stage, providing XGBoost with a compact and informative feature space. This reduces search complexity and overfitting risk during model training. Meanwhile, XGBoost further strengthens interaction learning among features through recursive tree splitting and weighted residual updates, enabling the model to fully exploit latent relationships between DBH and multi-dimensional information derived from height, canopy structure, and intensity features. This coupling mechanism allows the model to achieve improved prediction stability and generalization performance while maintaining reasonable interpretability.

Compared with existing studies, the proposed framework exhibits greater methodological coherence in feature selection and modeling. For example, Zhang et al. [60] applied random forest-based feature ranking followed by GBM modeling; however, their feature optimization relied on a single importance metric. In contrast, the MSFS framework integrates multiple selection criteria across linear, nonlinear, and importance-based dimensions, thereby overcoming the limitations of single-method feature evaluation. The MSFS–XGBoost framework not only provides a novel modeling strategy for high-dimensional LiDAR features but also establishes a methodological foundation for integrated modeling under “high-dimensional input–efficient learning” conditions in forest structural parameter estimation.

Despite its strong performance, the MSFS–XGBoost approach has several limitations. First, model performance is influenced by point cloud density and stand structural complexity. Under sparse point cloud conditions, the extraction accuracy of certain structural features (e.g., canopy density or crown-related metrics) may decrease, potentially introducing bias into model inputs [61,62,63]. Second, this study relies exclusively on airborne LiDAR data acquired from a single forest farm and focuses on two dominant tree species (*Cupressus lusitanica* and *Canarium oleosum*). Although the model demonstrates stable performance across these two species, the generalizability of the proposed framework to other forest types, species compositions, and stand conditions remains uncertain. In more complex or heterogeneous forest environments, the relationships between LiDAR-derived features and DBH may vary. Such variations may lead to distribution shifts in feature space, which can affect the stability of learned relationships and reduce model transferability across different ecological conditions. In addition, variations in LiDAR sensor types, acquisition parameters (e.g., flight altitude and scanning configuration), and point cloud density may influence feature extraction quality and model transferability. Therefore, when applying the MSFS–XGBoost framework to different ecological contexts or datasets, re-calibration of model parameters and re-selection of optimal features may be required.

Future research should focus on validating the proposed framework across diverse forest ecosystems and multi-source LiDAR datasets. Furthermore, integrating terrestrial laser scanning (TLS) or other remote sensing data sources may further enhance model robustness and spatial transferability. Overall, the MSFS–XGBoost framework demonstrates strong adaptability and practical potential under high-dimensional LiDAR feature conditions, providing an effective technical pathway for rapid estimation and dynamic updating of individual-tree structural parameters in forest resource monitoring and carbon stock assessment.

## 6. Conclusions

This study proposes an integrated individual-tree DBH estimation approach based on airborne LiDAR data by combining Multi-Stage Feature Selection with Extreme Gradient Boosting (MSFS–XGBoost), and systematically evaluates its performance and estimation accuracy. The results demonstrate that the proposed MSFS strategy effectively compresses the original set of 104 high-dimensional point cloud features into 13 core variables by jointly applying Pearson correlation analysis, Mutual Information (MI), and the Boruta algorithm. This process substantially reduces feature redundancy and multicollinearity while enhancing the representativeness and stability of the feature space. Comparative analyses of DBH estimation models indicate that MSFS–XGBoost consistently outperforms the other models across different feature sets. In particular, when using the final MSFS-optimized feature subset, the proposed model achieves a coefficient of determination (R^2^) of 0.901 and a root mean square error (RMSE) of 1.647, representing an improvement of 0.384 in R^2^ and a reduction of 1.146 in RMSE compared with the model built using the original feature set. These results clearly confirm the substantial contribution of feature optimization to enhancing the predictive performance of the XGBoost model. Furthermore, species-specific validation results show that the MSFS–XGBoost model maintains high accuracy and robustness for both *Cupressus lusitanica* and *Canarium oleosum*, demonstrating strong adaptability across different forest types. This indicates that the proposed framework exhibits good generalization capability at the individual-tree level.

Overall, this study highlights the critical role of feature selection in airborne LiDAR-based DBH estimation and demonstrates the advantages of integrating feature optimization with nonlinear ensemble learning. The proposed MSFS–XGBoost framework provides an efficient, reliable, and scalable approach for individual-tree structural parameter estimation, with strong potential for application in forest resource monitoring, carbon stock assessment, and smart forestry management. Future research may further enhance model performance and generalization by incorporating multi-source remote sensing data and multi-scale information.

## Figures and Tables

**Table 1:** Technical specifications of the UAV–LiDAR system.

**Zhihang SF1650 Hexacopter UAV**	
Parameter	Specification
Maximum take-off weight	21 kg
Maximum payload	6 kg
Maximum flight speed	18 km/h
Maximum control range	10 km
RTK positioning accuracy	(horizontal) 1 cm ± 1 ppm
RTK positioning accuracy	(vertical) 2 cm ± 1 ppm
**South Surveying & Mapping SAL-1500 LiDAR Sensor**	
Parameter	Specification
Weight	1 kg
Measurement range	1.5–1500 m
System accuracy	±5 cm
Maximum measurement rate	2 million points/s
Field of view	360°
Scanning angle	(horizontal) 80° × (vertical) 4.5°
Scanning frequency	200 kHz

**Table 2:** Summary of field-measured structural parameters.

Forest Type	Broadleaf Forest		Coniferous Forest	
No. of Plots	11		40	
Parameter	Range	Mean	Range	Mean
DBH (cm)	6.2–51.0	28.2	9.7–64.3	33.5
Tree Height (m)	5.1–25.6	11.2	6.2–22.6	15.7
Crown Diameter (m)	0.8–12.9	6.8	0.8–11.8	5.2
Crown Area (m^2^)	0.6–118.8	37.4	0.5–108.7	23.8

**Table 3:** Summary of extracted point cloud feature variables.

**Height Features (46)**			
Variable Name	Abbreviation	quantity	Variable Description
Average absolute deviation	E-aad	1	$V=\frac{\sum_{i=1}^{n}\left|Z_{i}-\bar{Z}\right|}{n}$
Canopy relief rate	E-crr	1	$V=\frac{Z_{mean}-Z_{min}}{Z_{max}-Z_{min}}$
Accumulate height percentiles	E-AIH	15	${\mathrm{AIH}}_{X\%}=\sum_{i=0}^{X\%}Z_{i}\left(X\;=1,\;5,\;10,\;20,\;25,\;30,\;40,\;50,\;60,\;70,\;75,\;80,\;90,\;95,\;99\right)$
Interquartile range of accumulate height percentile	E-AIH-IQ	1	$AIH_{IQ}={AIH}_{75\%}-{AIH}_{25\%}$
Variable coefficient	E-cv	1	$V=\frac{Z_{std}}{Z_{mean}}\times 100\%$
Kurtosis	E_kurtosis	1	$Kurtosis=\frac{\frac{1}{n-1}\sum_{i=1}^{n}{\left(Z_{i}-\bar{Z}\right)}^{4}}{\sigma^{4}}\;$
Median of median absolute deviation	E-Mad median	1	The median absolute deviation of the median height value at all points in the region.
Maximum, minimum, mean, median, skewness, standard deviation, and variance	E-max,E-min,E-mean, E-median,E-skewness, E-stddev,E-var	7	The maximum, minimum, mean, median, skewness, standard deviation, and variance of all point heights in the region.
Quadratic power mean	E-sms	1	$V=\sqrt{\frac{\sum_{i=1}^{n}Z_{i}^{2}}{n}}$
The mean to the third power	E-cmc	1	$V=\sqrt[{3}]{\frac{\sum_{i=1}^{n}Z_{i}^{3}}{n}}$
Percentile of height	E-P	15	$Elev=Z_{X\%}\;\left(X\;=1\;,5\;,10,\;20,\;25,\;30,\;40,\;50,\;60,\;70,\;75,\;80,\;90,\;95,\;99\right)$
Interquartile range of Percentile of height	E-PIQ	1	$V={Elev}_{75\%}-{Elev}_{25\%}$
**Intensity features (42)**			
Mean absolute deviation	I-aad	1	$V=\frac{\sum_{i=1}^{n}\left|I_{i}-\bar{I}\right|}{n}$
Accumulate intensity percentiles	I-AII	15	${\mathrm{AII}}_{X\%}=\sum_{i=0}^{X\%}I_{i}\left(X=1,5,10,20,25,30,40,50,60,70,75,80,90,95,99\right)$
Variable coefficient	I-cv	1	$V=\frac{I_{std}}{I_{mean}}\times 100\%$
Kurtosis	I_kurtosis	1	$Kurtosis=\frac{\frac{1}{n-1}\sum_{i=1}^{n}{\left(I_{i}-\bar{I}\right)}^{4}}{\sigma^{4}}\;$
Median of median absolute deviation	I-Mad median	1	The median absolute deviation of the median intensity value at all points in the region.
Percentile of intensity	I-P	15	$Int=I_{X\%}\;\left(X\;=1,\;5,\;10,\;20,\;25,\;30,\;40,\;50,\;60,\;70,\;75,\;80,\;90,\;95,\;99\right)$
Interquartile range of percentile of intensity	I-PIQ	1	$V={Int}_{75\%}-{Int}_{25\%}$
Maximum, minimum, mean, median, skewness, standard deviation, and variance	I-max,I-min,I-mean,I-median,I-skewness,I-stddev, I-var	7	The maximum, minimum, mean, median, skewness, standard deviation, and variance of intensity values of all points in the region.
**Density and other features (13)**			
Density	D-M	10	In the region, the point cloud data are divided into ten equal height slices from low to high and the proportion of echo numbers in each layer is the corresponding density variable.
Canopy cover	CC	1	$CC=\frac{n_{veg}}{n_{total}}$ ($n_{veg\;}$ is the number of vegetation points, $n_{total}$ is the total number of points)
Leaf area index	LAI	1	$LAI=\frac{cos\left(ang\right)\times In\left(GF\right)}{k}$ (ang is the average scan Angle, GF is the gap rate, and k is the extinction coefficient)
Gap Fraction	GF	1	$GF=\frac{n_{ground}}{n}$ ($n_{ground\;}$ is the number of ground points whose height is lower than the height threshold and n is the total number of points)

**Table 4:** Overview of regression models used for comparison.

Model	Basic Principle	Advantages	Representative Significance
DTR [50]	Constructs a tree structure through recursive feature splitting and predicts target values at leaf nodes	Simple structure; easy to interpret	Serves as a baseline model for comparison with ensemble methods
RFR [51]	Integrates multiple decision trees using bootstrap sampling and voting/averaging to reduce variance	High stability; strong resistance to noise	Represents bagging-based ensemble methods; suitable for high-dimensional point cloud data
GBM [52]	Iteratively learns residuals to progressively improve the performance of weak learners	High fitting accuracy; capable of modeling complex relationships	Represents boosting-based methods and evaluates their regression performance

**Table 5:** Accuracy assessment of individual-tree segmentation.

Forest Type	Actual Number	Segmented Number	TP	FN	FP	R	P	F
Coniferous forest	586 (trees)	635 (trees)	532	54	133	0.91	0.80	0.85
Broadleaf forest	150 (trees)	161 (trees)	138	12	24	0.92	0.85	0.88

**Table 6:** Feature subsets retained at different stages of the MSFS procedure (numbers in parentheses indicate feature counts).

Method	Category	Variable Names
Pearson (60 features retained)	Structural parameters (3)	Tree height, Crown diameter, Crown area
	Height features (38)	E_min, E_crr, E_P90, E_P95, E_AIH95, E_AIH99, E_AIH90, E_P99, E_P80, E_P75, E_P70, E_AIH80, E_ cv, E_ stddev, E_AIH75, E_ max, E_P60, E_ cmc, E_AIH70, E_P50, E_ sms, E_ aad, E_AIH60, E_P40, E_ mean, E_AIH50, E_ median, E_P30, E_AIH_IQ, E_P20, E_P25, E_AIH40, E_kurtosis, E_IQ, E_P10, E_AIH30, E_AIH25, E_ mad median
	Intensity features (18)	I_ max, I_P30, I_P40, I_cv, I_P25, I_AII5, I_P50, I_ median, I_AII1, I_P20, I_AII10, I_P60, I_ mean, I_AII99, I_P70, I_P99, I_AII95, I_P75
	Density features (1)	D_M [5]
MI (30 features retained)	Structural parameters (3)	Tree height, Crown diameter, Crown area,
	Height features (16)	E_P80, E_crr, E_cv, E_AIH90, E_AIH80, E_P40, E_AIH95, E_P75, E_P50, E_P90, E_min, E_AIH70, E_P70, E_AIH75, E_P60, E_ cmc,
	Intensity features (11)	I_P25, I_P60, I_ cv, I_P70, I_ max, I_P99, I_P75, I_ median, I_P50, I_AII95, I_AII10
Boruta (13 features retained)	Structural parameters (3)	Tree height, Crown diameter, Crown area
	Height features (5)	E_min, E_crr, E_cv, E-AIH90, E-P75
	Intensity features (5)	I-cv, I-max, I-P99, I-AII95, I-AII10

**Table 7:** Statistics of retained feature set selected by the Boruta algorithm.

Category	Variable Names	Correlation Coefficient	Importance Index	Significance Level
Structural parameters	Tree height	0.756	0.823	0.000
	Crown diameter	0.724	0.781	0.000
	Crown area	0.689	0.756	0.000
Height features	E_min	0.664	0.689	0.000
	E_crr	0.693	0.718	0.000
	E_cv	0.542	0.561	0.015
	E-AIH90	0.612	0.635	0.010
	E-P75	0.578	0.597	0.010
Intensity features	I-cv	0.523	0.548	0.000
	I-max	0.681	0.697	0.000
	I-P99	0.635	0.658	0.001
	I-AII95	0.574	0.593	0.010
	I-AII10	0.653	0.673	0.000

**Table 8:** Statistics of individual-tree samples used for modeling and validation.

Tree Species	Number	Train Set			Test Set		
		Number	DBH/cm	Mean/cm	Number	DBH/cm	Mean/cm
*Cupressus* *lusitanica*	500	333	5.1–60.8	28.8	167	6.8–65.1	30.9
*Canarium* *oleosum*	100	67	6.2–48.0	21.5	33	8.4–45.3	19.8

**Table 9:** Comparison of DBH estimation accuracy among different regression models.

Model	Dimension	Max-Depth	n-Estimators	Train Set		Test Set	
				R^2^	RMSE	R^2^	RMSE
DTR	104	5	—	0.365	4.090	0.330	4.121
	60	5	—	0.412	4.120	0.395	4.468
	30	7	—	0.528	3.912	0.490	4.027
	13	7	—	0.603	3.824	0.553	3.850
GBM	104	3	50	0.478	2.889	0.443	3.314
	60	3	50	0.629	2.651	0.577	2.860
	30	5	110	0.675	2.524	0.619	2.528
	13	7	170	0.835	1.975	0.783	2.049
RFR	104	5	50	0.391	3.912	0.358	4.027
	60	7	90	0.598	4.050	0.550	4.243
	30	7	100	0.649	3.535	0.661	3.658
	13	9	150	0.711	3.031	0.693	3.248
XGBoost	104	3	250	0.558	2.651	0.517	2.793
	60	3	150	0.731	2.355	0.679	2.389
	30	3	200	0.875	1.928	0.820	2.030
	13	3	250	0.916	1.501	0.901	1.647

**Table 10:** Cross-validation results of different models using the optimal feature set.

Model	Dimension	Max-Depth	n-Estimators	R^2^ (Mean ± Std)	RMSE (Mean ± Std)
DTR	13	7	—	0.533 ± 0.022	3.880 ± 0.043
RFR	13	9	150	0.673 ± 0.023	3.288 ± 0.045
GBM	13	7	170	0.763 ± 0.019	2.079 ± 0.036
XGBoost	13	3	250	0.881 ± 0.016	1.677 ± 0.031

**Table 11:** Comparison of feature selection methods for DBH estimation using the XGBoost model.

Method	Dimension	Max-Depth	n-Estimators	R^2^	RMSE (cm)
MSFS	13	3	250	0.901	1.647
LASSO	20	3	250	0.814	2.219

**Table 12:** Species-specific evaluation of model performance.

Tree Species	Model	Dimension	Max-Depth	n-Estimators	R^2^	RMSE
*Cupressus lusitanica*	XGBoost	13	3	250	0.905	1.518
	GBM	13	7	170	0.813	2.096
	RFR	13	9	150	0.675	3.025
	DTR	13	7	none	0.537	3.621
*Canarium oleosum*	XGBoost	13	3	250	0.896	1.614
	GBM	13	7	170	0.782	2.167
	RFR	13	9	150	0.691	2.959
	DTR	13	7	none	0.553	3.855

## Data Availability

The data presented in this study are available on request from the corresponding author.
